# Pulmonary toxicity after intraperitoneal mitomycin C: a case report of a rare complication of HIPEC

**DOI:** 10.1186/s12957-016-1047-6

**Published:** 2017-02-20

**Authors:** Melissa L. Abel, George Kokosis, Dan G. Blazer

**Affiliations:** 1 School of Medicine, Duke University Medical Center, Box 3247, Durham, NC 27710 USA; 2 Department of Surgery, Duke University Medical Center, Box 3247, Durham, NC 27710 USA

**Keywords:** Appendiceal cancer, Intraperitoneal chemotherapy, Mitomycin C, Pulmonary toxicity, ARDS

## Abstract

**Background:**

Cytoreductive surgery combined with hyperthermic intraperitoneal chemotherapy (CRS/HIPEC) has become a common treatment approach for disseminated appendiceal neoplasms. Systemic absorption of intraperitoneal chemotherapeutics may lead to drug-induced toxicity, most commonly neutropenia. Mitomycin C has been the most commonly used chemotherapeutic in HIPEC for the past several decades.

**Case presentation:**

Here, we describe a rare pulmonary complication secondary to intraperitoneal administration of mitomycin C.

**Conclusions:**

While rare, intraperitoneal mitomycin C has the potential to cause serious pulmonary toxicity that should be considered with administration. To our knowledge, this report represents only the second case described in the literature.

## Background

Aggressive surgical approaches to patients with peritoneal carcinomatosis from nongynecologic malignancies have become increasingly common, abrogating some of the historic nihilism associated with treating this difficult patient population [1]. Specifically, over the last three decades, cytoreductive surgery combined with hyperthermic intraperitoneal chemotherapy (CRS/HIPEC) has emerged as a viable treatment option for selected patients with reasonable morbidity and favorable oncologic outcomes [1, 2].

In patients with disseminated colorectal and appendiceal malignancies undergoing CRS/HIPEC, mitomycin C (MMC) has been the preferred chemotherapeutic agent for decades [3–6]. It is a large molecule with limited systemic absorption, it rapidly penetrates tumor cells, and it is synergistic with hyperthermia [7, 8]. The most common toxicity associated with MMC in HIPEC is neutropenia, which has been shown to occur in up to 39 % of patients [9, 10]. Intravenous MMC is well-known to cause dose-dependent interstitial lung disease, but reports of pulmonary toxicity secondary to intraperitoneal administration are rare [11–13]. Here, we present a case of acute respiratory distress syndrome (ARDS) secondary to MMC administration in a patient recovering from CRS/HIPEC for disseminated appendiceal cancer.

## Case presentation

A 38-year-old female with an unremarkable past medical history initially presented to an outside facility with acute-onset low back pain. MRI showed a fluid-filled appendix, and a subsequent CT scan raised concern for acute appendicitis. A laparoscopic appendectomy was performed on the 11th of August 2015 with intraoperative findings of a swollen appendix without any evidence of rupture. Frozen section of the specimen revealed at least low-grade dysplasia with negative margins, and the procedure was terminated at that point. Final pathology revealed invasive adenocarcinoma with evidence of perforation and normal mesoappendix making this a T4Nx tumor. Peritoneal washings revealed tumor cells present.

The patient was then referred to our institution for consideration of CRS/HIPEC. After multidisciplinary tumor board discussion, the patient underwent 3 months of XELOX therapy with plans for interval CRS/HIPEC. In January 2016, the patient underwent laparoscopic right hemicolectomy, omentectomy, and HIPEC. Prior to incision, 1 g of ertapenem was given per routine. No gross disease was appreciated on exploration. The placement of the HIPEC cannulae was performed through the laparoscopic extraction site of the colon and omentum, essentially facilitating a minimally invasive HIPEC. Per institutional practice, HIPEC involved administration of 40 mg MMC with a target intraperitoneal temperature of 41 °C for 90 min. Per institutional protocol, mitomycin C is given at a fixed dose and not dosed by body surface area. Thirty milligrams is administered at time 0 min, and 10 mg administered at time 60 min. Total perfusate is 3 L of normal saline.

During the operation, the patient received 2.2 L of total fluid, primarily lactated Ringer’s solution, over the course of 5 h. No blood products were given, and the patient made 265 mL of urine during the case with an estimated blood loss of 50 mL. The patient’s fluid balance over the next 24 h was essentially even, 2.5 L of saline and 1.8 L of urine output recorded. No blood products were given postoperatively either.

**Fig. 1:**
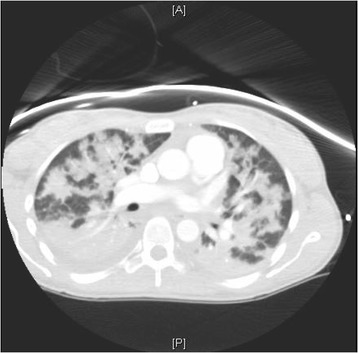
Chest CT with IV contrast obtained at the onset of respiratory insufficiency, showing new diffuse bilateral heterogeneous and consolidative opacities with small right greater than left pleural effusions, consistent with ARDS

On postoperative day 2, the patient developed acute respiratory distress with increasing oxygen requirements. She was febrile to 39.5 ° C and acutely tachycardic, with a heart rate of 143 bpm. She was placed on a partial rebreather and transferred to the surgical ICU. A CT scan was obtained, which ruled out pulmonary embolism but showed marked edema and infiltration of the lungs (Fig. 1). The patient was started on vancomycin/piperacillin-tazobactam/azithromycin for presumed pneumonia. She was given 20 mg furosemide intravenously with excellent diuresis. She received an additional dose on postoperative day 3. Nasal swab for respiratory syncytial virus and *Legionella* and *Strep pneumoniae* urine antigen studies were sent and ultimately returned as negative, and WBCs were within normal limits throughout this event. Additional studies including sputum and blood cultures were all negative.

**Fig. 2:**
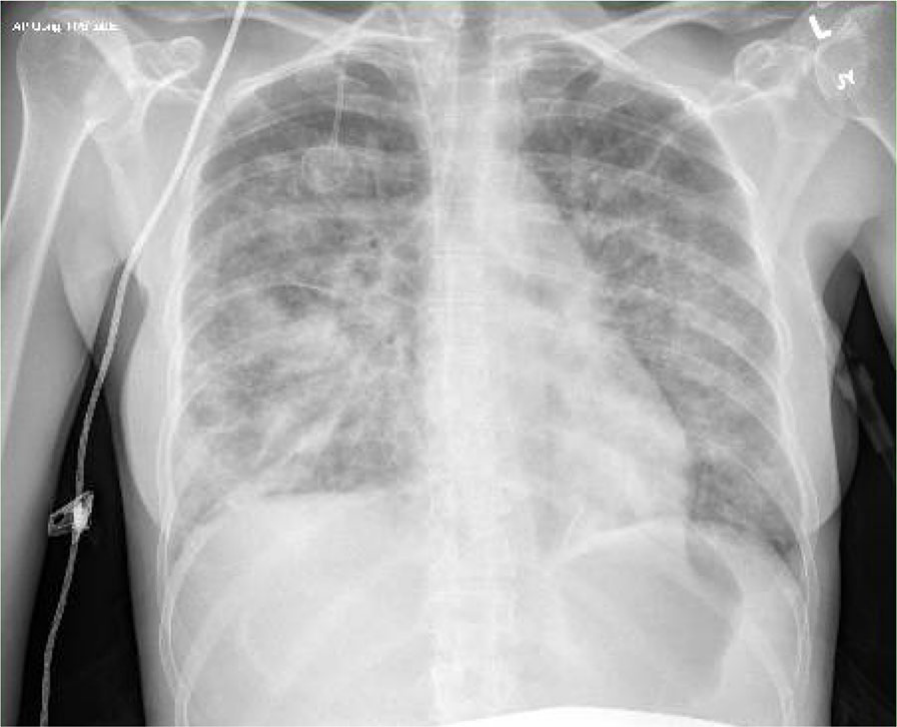
Frontal projection chest X-ray taken at onset of respiratory insufficiency, showing diffuse bilateral heterogeneous opacities

On postoperative day 4, the patient’s respiratory status continued to worsen despite the use of intermittent bilateral positive airway pressure (BiPAP), ultimately requiring intubation. Phenylephrine was also administered for blood pressure support. At this time, chest X-ray showed increasing pulmonary opacities (Fig. 2). The pulmonology service was consulted on postoperative day 5, and their team concluded that this patient had ARDS of uncertain etiology given negative infectious workup to date. Bronchoscopy was recommended if the patient failed to improve.

**Fig. 3:**
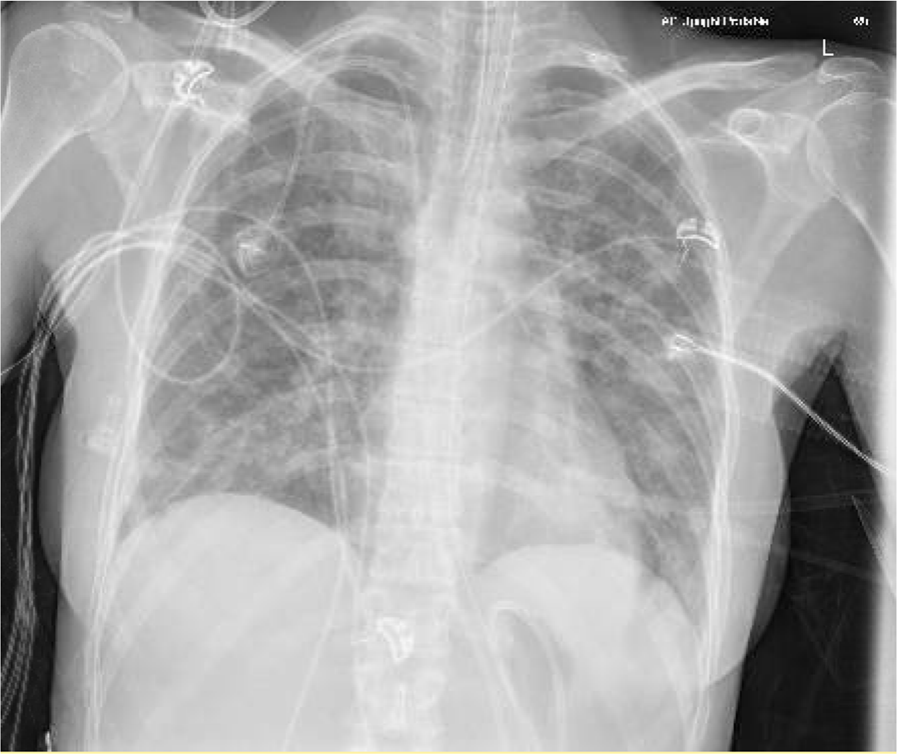
Repeated chest X-ray 5 days later, with improving bilateral opacities

However, following intubation, the patient rapidly improved and was extubated by postoperative day 7. Though she remained essentially afebrile from postoperative day 2 until discharge (Tmax no greater than 38 C), empiric antibiotics were continued until discharge, with discontinuation of azithromycin on postoperative day 7. Repeated chest X-ray was obtained and showed marked improvement (Fig. 3). The patient continued to improve with no complications following extubation and was discharged home on postoperative day 10 without need of supplemental oxygen, tolerating a regular diet, and with return of bowel function.

At no point during the postoperative course was there ever any evidence of abdominal sepsis to explain the pulmonary findings. Throughout her postoperative course, her abdomen remained appropriately soft; she had an early return of bowel function, with no clinical evidence of peritonitis. Furthermore, an abdominal CT scan done at the onset of respiratory insufficiency on postoperative day 2 showed normal postsurgical changes, but no fluid collection or other evidence of anastomotic leak, making an abdominal source for her pulmonary toxicity unlikely.

The patient experienced mild neutropenia 5 days following the onset of respiratory distress, which resolved within a few days. Prior to her operation, the patient had a white blood cell count of 3.9 × 10^9^ cells/L, which increased within the normal range to 7.9 × 10^9^ at the onset of respiratory insufficiency. Her white blood cell count then progressively decreased to a mild neutropenia, reaching 2.6 × 10^9^ by postoperative day 7. At the time of her discharge her neutropenia had resolved, with a count of 8.0 × 10^9^.

At the patient’s most recent follow-up appointment, over 1 month postoperatively, she reported no shortness of breath or chest pain, with an oxygen saturation of 99 % on room air with a respiratory rate of 16. Overall, she is doing well from a respiratory standpoint. She is scheduled to restart systemic chemotherapy.

## Conclusions

Cytoreductive surgery in combination with HIPEC is a well-accepted and increasingly utilized treatment strategy for patients with disseminated appendiceal malignancies. MMC is the most frequently used chemotherapeutic agent for this approach, in part due to its high molecular weight that allows for limited systemic penetration [13]. Unlike conventional systemic chemotherapy for appendiceal cancer, which has limited access to the peritoneum, MMC HIPEC allows for high local doses targeted at residual micrometastatic peritoneal disease [7, 14]. The hyperthermia used in HIPEC synergizes with the antitumor effects of intraperitoneal chemotherapy by increasing cytotoxicity as well as the depth of penetration by the drug [7].

Although this strategy has favorable oncologic outcomes compared to systemic chemotherapy alone, CRS/HIPEC carries potential for significant adverse effects. Morbidity following this treatment is most commonly related to cytoreductive surgery; yet, there remains a risk for MMC-related toxicity [13]. The most frequent side effect from peritoneal MMC is neutropenia, for which female sex and MMC dose per body surface area have been implicated as risk factors [9]. The patient presented in this case, although she had a significant drop in white blood cell count resulting in a Grade 2 leukocyte toxicity (from 7.2 × 10^9^ to 2.5 × 10^9^), did not experience neutropenia (ANC 2.2 × 10^9^) [15]. Of note, she also had a significant drop in her hemoglobin to a Grade 3 anemia (10.4 to 7.3 g/dL) without significant blood loss [15].

More notable and unique in this case was the onset of respiratory symptoms after treatment with MMC. This patient was ultimately diagnosed with ARDS beginning 2 days post-therapy due to a constellation of factors that include acute-onset respiratory insufficiency with an FiO2/paO2 of 250, bilateral pulmonary infiltrates on imaging, and lack of cardiogenic pathology. The presence of a degree of myelosuppression in this patient, as evidenced by the drop in her ANC and hematocrit postoperatively, confirms an element of systemic absorption of MMC, which supports the diagnosis of MMC toxicity-induced ARDS. Given the negative infectious workup, a noninfectious cause for ARDS in this patient was strongly favored. The strong temporal relationship with administration of intraperitoneal mitomycin C and development of pulmonary toxicity also favors drug toxicity as the inciting cause for ARDS. Given the well-established relationship between systemic mitomycin C and development of interstitial pneumonitis, the pulmonary and critical care teams strongly favored this diagnosis. However, given the patient’s relatively rapid recovery, no lung biopsy was performed and empiric steroid therapy was never instituted.

A recent study on the neutropenic effects of MMC HIPEC resulted in a standardized, weight-based algorithm dosing system adjusted for the presence of prior systemic chemotherapy to minimize neutropenia [9]. This patient received a dose of MMC HIPEC consistent with this algorithm. While she did not develop neutropenia, we believe that the development of ARDS in addition to an evidence of myelosuppression is both attributable to intraperitoneal administration of MMC. Unique to this patient is her BMI being <20 (19.3) and her body surface area being only 1.61 m^2^. Side effects of MMC HIPEC are reported to happen at a median BMI of 25.5 (range 19–36.2) and BSA of 1.77 (range 1.39–2.36) [9].

The first cases of interstitial pneumonitis during treatment with intravenous MMC were reported in 1978 [16]. Following this publication, pulmonary toxicity continued to be reported as a rare but well-known side effect of MMC, with occurrence rates of 5 to 12 % [17]. The first prospective study on the relationship between intravenous MMC and pulmonary toxicity concluded that this toxicity is a dose-dependent side effect of MMC that should be considered only when patients receive over a 20 mg/m^2^ cumulative dose [18]. This model was supported by later pharmacological evidence that there is a direct relationship between body surface area and MMC plasma clearance, as well as between plasma exposure and hematological toxicity [14]. Recommendations of this study were to use a dose of 25 mg/m^2^, divided into three portions of 50 % at the beginning of treatment, 25 % after 30 min, and 25 % after another 30 min, in order to limit the incidence of leukopenic side effects to 10 %.

Despite the established relationship between intravenous MMC and interstitial pneumonitis, large studies of MMC HIPEC-induced pulmonary toxicity have yet to be reported in the literature. A 2008 report outlined the first published case of HIPEC MMC-induced interstitial pneumonitis in a patient who received 30.8 mg/m^2^ HIPEC MMC within the standard treatment dosing parameters [13]. This case similarly demonstrated systemic penetration of MMC despite dosing according to established guidelines. Unlike the patient presented here, however, the previous case describes a case of interstitial pneumonitis 37 days following therapy, requiring steroids but without need for intubation. Both accounts draw attention to the potential for respiratory toxicity associated with HIPEC MMC.

It remains unclear what, if any, underlying factors may have predisposed these patients to acquire respiratory insufficiency, but the existence of such factors could/may help providers better predict which patients are vulnerable to these side effects. Although rare, it is clear that oncologists must be aware of the potential for pulmonary toxicity of MMC, which we have seen possible with either intravenous or intraperitoneal administration. These events should be monitored for and treated aggressively, and further cases must continue to be reported to bring insight to the mechanisms that underlie this process, which patients may be at increased risk, and how dosing of HIPEC MMC might be altered to avoid further complications.

## Abbreviations

ANC: Absolute neutrophil count
ARDS: Acute respiratory distress syndrome
CRS/HIPEC: Cytoreductive surgery combined with hyperthermic intraperitoneal chemotherapy
IP: Intraperitoneal
MMC: Mitomycin C.
